# Coronary artery bypass grafting and paraparesis;is there a correlation?

**DOI:** 10.5830/CVJA-2017-014

**Published:** 2018

**Authors:** Samiotis Ilias, G Baikoussis Nikolaos, Patris Vasileios, Argiriou Michalis, Dedeilias Panagiotis, Charitos Christos

**Affiliations:** Cardiovascular and Thoracic Surgery Department, Evangelismos General Hospital of Athens, Athens, Greece; Cardiovascular and Thoracic Surgery Department, Evangelismos General Hospital of Athens, Athens, Greece; Cardiovascular and Thoracic Surgery Department, Evangelismos General Hospital of Athens, Athens, Greece; Cardiovascular and Thoracic Surgery Department, Evangelismos General Hospital of Athens, Athens, Greece; Cardiovascular and Thoracic Surgery Department, Evangelismos General Hospital of Athens, Athens, Greece; Cardiovascular and Thoracic Surgery Department, Evangelismos General Hospital of Athens, Athens, Greece

**Keywords:** coronary artery bypass grafting, CABG, stroke, extracorporeal circulation, neurological complications in cardiac surgery, paraparesis, spinal cord ischaemia, transient ischaemic attack

## Abstract

Adult cardiac surgery is associated with significant perioperative morbidity and mortality rates, mainly in elderly patients with co-morbidities. A series of postoperative complications may arise and delay the recovery of patients undergoing cardiac surgery. Such complications also increase the burden of resource use and may affect late survival rates. Neurological complications appear mainly as stroke of varying degrees, with impairment of mobility and ability of the patient. We describe a rare case of progressive paraparesis after on-pump coronary artery bypass grafting, and review its aetiology, diagnosis and management.

Neurological complications after cardiac surgery may occur in the post-operative period. Stroke and transient ischaemic attack are major adverse cardiac events following coronary artery bypass grafting (CABG) and markedly reduce patient shortand long-term survival rates. The causes of these complications are hypoxia, metabolic abnormalities, emboli or haemorrhage. These complications are associated with increased mortality rates, prolonged intensive care unit (ICU) stay and decreased long-term survival rates.^1^-^7^ The main risk factors for neurological complications in cardiac surgery are haemodynamic instability, diabetes mellitus, advanced age, complex procedures, prolonged cardiopulmonary bypass time (CPB > two hours), previous stroke,hypertension, hyperglycaemia, hyperthermia, hypoxaemia, aorticatheromatosis and peripheral vascular disease.^1^,^2^,^5^

## Case report

A 65-year-old man was admitted to our department for a routine CABG due to left main coronary artery disease. The patient’s medical history included smoking, family history of early coronary artery disease, hypertension, diabetes, hyperlipidaemia, percutaneous transluminal coronary angioplasty to the left descending artery (LAD) and to the right coronary artery (RCA) 12 years earlier, and myocardial infarction 11 years earlier due to in-stent stenosis. In his past medical history, there was an unclear history of sensory or motor impairment after coccyx cyst surgery.

All laboratory data were within normal limits except for the erythrocyte sedimentation rate (521st, 1 132nd) and a C-reactive protein (CRP) > 2 mg/l. Echocardiographic findings were left ventricular ejection fraction (LVEF) of 45% and mild left ventricular hypertrophy. Coronary artery CT-angiography was performed and stenosis of three coronary arteries was established.

The induction of anaesthesia was performed with Dormicum 5 mg, Propofol 150 mg, Esmeron 60 mg and Sevoflurane. The patient underwent triple coronary artery bypass grafting as follows: left internal mammary to left anterior descending artery (LIMA–LAD), a saphenous vein graft to the first obtuse marginalis (SVG–OM1) and another saphenous vein graft to the right coronary artery (SVG–RCA). During surgery his vital signs were stable and the arterial blood gasses (ABGs) were within normal limits.

After surgery the patient was moved to the cardiac ICU while intubated and unconscious, with a blood pressure of 110/60 mmHg, heart rate of 77 beats/min and normal sinus rhythm, central venous pressure of 8 cm H_2_O and peripheral capillary oxygen saturation of 100%. After admission to the ICU, his primary vital signs were normal. The patient was successfully weaned and extubated on the same day. The post-surgery drugs were: enoxaparin 40 mg daily, furosemide 20 mg daily, metoprolol 100 mg twice daily, clopidogrel 75 mg daily, atorvastatin 20 mg daily and acetylsalicylic acid 100 mg daily.

On the first postoperative day, laboratory findings in the ICU were: haemoglobin 10 g/dl, haematocrit 30.5%, platelets = 242 000 cells/l, white blood cell count = 9 100 cells/l, prothrombin time = 15.3 sec, activated partial thromboplastin time = 32 sec, INR = 1.47, sodium = 139 mEq/l, potassium = 4.9 mmol/l, blood urea nitrogen (BUN) = 17 mg/dl, creatinine = 0.94 mg/dl, creatine phosphokinase (CK) = 847 U/l, CK-MB = 58 U/l. He was moved to the cardiac surgery department. During this first postoperative day, the patient was stable, awake and oriented. No signs of haemodynamic instability or cardiac dysrhythmias were seen.

The second day after CABG, the overall condition of the patient was good but he had difficulty moving his lower limbs. Neurological consultation was done and the cranial nerves were found to be intact, cerebellar tests and sensory examinations were normal, muscle strength of the lower limbs was 3/5 and symmetric and plantar reflexes were double flexor.

On the third postoperative day, the overall condition of the patient was good but he still had difficulty moving the lower limbs. Progressive paraparesis developed and the muscle strength and deep tendon reflexes (DTRs) began to decrease gradually. Paraparesis progressed and muscle strength decreased from 4/5 to 3/5 and then to 2/5. In the evening, severe weakness of the lower and upper limbs developed, absence of DTRs, no plantar reflexes, and muscle strength was 1/5 on the left and 0/5 on the right side. That night the patient presented with respiratory failure; he was intubated and moved to the ICU.

On the fourth day, the patient was haemodynamically stable and he was transferred to the radiology laboratory in order to undergo magnetic resonance imaging (MRI). During the MRI examination, the patient experienced an episode of ventricular fibrillation and cardiac arrest. He was resuscitated after 20 minutes of cardiopulmonary resuscitation and two defibrillations. He was in haemodynamic instability and received high doses of dobutamine, norepinephrine and adrenaline.

The laboratory findings were: creatinine = 3.0 mg/dl, urea 111 mg/dl, aspartate transaminase (AST) 1 000 U/l, alanine transaminase (ALT) 6182, LDH 9 119 U/l, CK 3 915 U/l, CK-MB 315 U/l, troponin 10 000 ng/ml. The echocardiogram findings were left atrium 39 mm, telo diastolic volume of the left ventricle 50 mm, the left ventricle showed diffuse hypokinesis and akinesis, with a LVEF of 25%, and pulmonary artery systolic pressure was 40–45 mmHg.

**Fig. 1:**
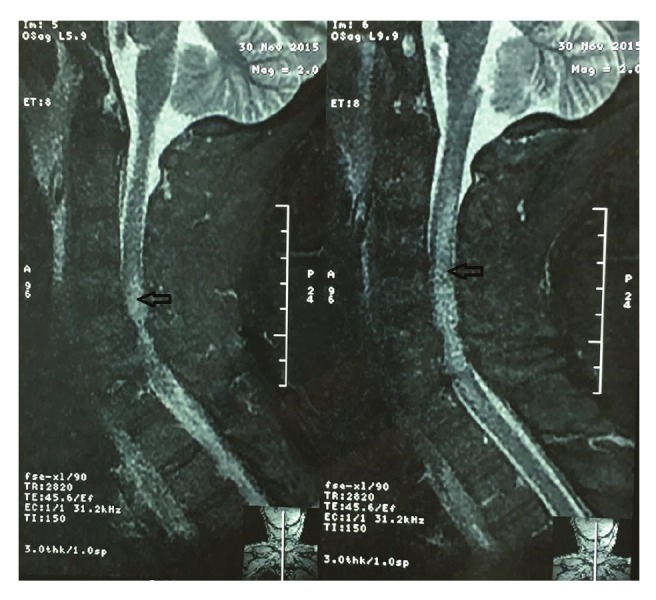
At the level of the fifth and sixth cervical vertebrae,posterior osteophytes and circular degeneration ofthe annulus fibrosis with high-grade stenosis andcompressive phenomena in the spinal cord can beseen. From the second to the sixth cervical level, apathological zone (white area into the grey zone) andoedema due to myelopathy and ischaemia (arrows)can be seen.

The MRI report showed at the level of the fifth and sixth cervical vertebrae that there were posterior osteophytes and circular degeneration of the annulus fibrosis with high-grade stenosis and compressive phenomena in the spinal cord. From the second to the sixth cervical vertebrae, there was a pathological zone and oedema due to myelopathy and ischaemia (Fig. 1).

On the sixth day after surgery, the patient was better and was haemodynamically supported with low-dose norepinephrine. However he presented manifestations of post-cardiac arrest brain injury such as coma, seizures and myoclonus. He died 10 days after surgery due to septic shock.

## Discussion

Neurological complications after cardiac surgery may occur in the post-operative period. Stroke and transient ischaemic attack are major adverse cardiac events following CABG and markedly reduce patient short- and long-term survival rates. The five-year rate of stroke was significantly higher after CABG than after percutaneous coronary intervention in patients with diabetes and multi-vessel coronary artery disease.^2^ Previous studies comparing surgical outcomes of patients undergoing conventional on-pump CABG with cardiopulmonary bypass versus patients undergoing off-pump CABG mostly focused on low-risk patients, for instance, relatively young patients with preserved left ventricular function and without systemic co-morbidity.

The causes of these neurological complications are hypoxia, metabolic abnormalities, emboli or haemorrhage. These complications can be divided into two types: type 1 (3%) includes major focal deficit and stupor or coma; type 2 (3%) includes intellectual dysfunction. These complications are associated with increased mortality rates, prolonged ICU stay and decreased long-term survival rates.^1^-^7^

Risk factors for neurological complications in cardiac surgery are haemodynamic instability, diabetes mellitus, advanced age, complex procedures, prolonged CPB (> two hours), previous stroke, hypertension, hyperglycaemia, hyperthermia, hypoxaemia, aortic atheromatosis and peripheral vascular disease.^1^,^2^,^5^ Mechanisms and factors causing neurological lesions are embolisation and hypoperfusion, and influencing factors are aortic atheroma plaque, cerebrovascular disease, altered cerebral autoregulation, hypotension, intracardiac debris air, venous obstruction on bypass, CPB circuit surface, cerebral hyperthermia and hypoxia.

In our case, the differential diagnoses were cerebrovascular accident or lacunar infarct (internal capsular region) – embolic or haemorrhagic, spinal cord ischaemia and infarct (due to embolic insult or hypoperfusion), acute inflammatory demyelinating polyradiculoneuropathy or critically ill polyneuropathy, peripheral nervous injury, peripheral vascular disease and spinal cord ischaemia. In spinal cord ischaemic stroke, neurological deficits may occur without pain, however, most (> 80%) are painful and this is an interesting difference from cerebral infarction, which is usually not painful. Depending on the level of the cord lesion, symptoms may vary from mild to moderate and even from reversible leg weakness to quadriplegia. Fever is a warning to consider infectious origins such as acute meningitis.^8^-^13^

Involvement of intrinsic cord vessels has been reported with arteritis such as systemic lupus erythematosus. Anterior spinal artery occlusion has been reported with arteritis, including that associated with syphilis and diabetes mellitus; after trauma; and as a complication of spinal angiography, spinal adhesive arachnoiditis, administration of intrathecal phenol, and spinal anaesthesia.

Aortic diseases are blamed for producing spinal infarction in a variety of situations including dissecting aneurysm; aortic surgery, especially with aortic cross-clamping above the renal artery; aortography; atherosclerotic embolisation; and aortic thrombosis. Uncommon causes include complications of abdominal surgery, particularly sympathectomy; circulatory failure as a result of cardiac arrest or prolonged hypotension; and vascular steal in the presence of an arteriovenous malformation, or vascular compression by tumours in the spinal canal, vertebral fracture, or a herniated intervertebral disk.^12^,^13^

Suspecting neuropathies, we ordered electromyography andnerve conduction velocity tests, which found peripheral sensory–motor polyneuropathy, mainly of the demyelinating type, witha differential diagnosis of acute inflammatory demyelinatingpolyneuropathy and critical illness polyneuropathy. A cervical–thoracic MRI showed the pathological osteophytes and circulardegeneration of the annulus fibrosis of the fifth and sixthcervical vertebra with a compression in the spinal cord andconsequent ischaemia and oedema of the medulla.

## Conclusion

The complication in our case is a rare condition that has not been discussed in the literature. We considered that this complication was due to a degenerative vertebral condition with compression in the spinal cord, exacerbated by placing a prop under the shoulder to position the body for the sternotomy. Placing a prop to position the body for CABG surgery is unavoidable and one cannot consider possible vertebral pathology in all patients before surgery, especially when the patient does not have any associated symptoms. Therefore in this patient, the diagnosis of paraparesis was made accidentally.
